# Supplementary material for: Integration of a (–Cu–S–)n plane in a metal–organic framework affords high electrical conductivity
Source: Nat Commun. 2019 Apr 12;10:1721. doi: 10.1038/s41467-019-09682-0 (PMC6461620; doi:10.1038/s41467-019-09682-0)
Supplement: Supplementary file 1 — Supplementary Information [file 41467_2019_9682_MOESM1_ESM.pdf]

Supplementary Information

Integration of a $(–Cu–S–)_n$ Plane in a Metal–Organic Framework Affords High Electrical Conductivity

Pathak, et al.

Supplementary Methods

Chemical reagents were purchased commercially and were used without further purification. Infrared spectra were obtained using a Perkin−Elmer Model Paragon 1000 FT-IR spectrometer in the range of 4000−400 cm^{-1}. Elemental analyses were performed on a Perkin−Elmer 2400 CHN elemental analyzer. Thermogravimetric (TG) analyses were done in an inert atmosphere of nitrogen using a Perkin−Elmer TGA-7 TG analyzer. A Perkin–Elmer Model Lambda 750 UV–vis spectrometer was used to collect diffuse reflection spectra (DRS). Finely powdered MOF was used as a sample and $BaSO_4$ as a reference to obtain DRS spectra. The Mercury 1.4.1 software program was used to produce simulated powder diffraction patterns. A Bruker-Nonius Kappa CCD diffractometer was used for the single crystal X-ray diffraction measurements. This diffractometer uses graphite monochromatic Mo Kα radiation with a wavelength of 0.7107 Å. Brunauer–Emmett–Teller analyses were carried out utilizing nitrogen gas as an adsorbent with a Micrometrics ASAP 2020 system at 77 K. The SADABS program was applied to correct for absorption. Direct methods were utilized to solve the crystal structure and were refined with the aid of the WINGX,[S1] PLATON,[S2] and SHELX[S3] software packages using the full-matrix least-squares on F^2 values. Independent anisotropic displacement parameters were utilized for the refinement of non-hydrogen atoms. The hydrogen atoms linked to carbon and nitrogen atoms were generated geometrically. A difference Fourier map was used to locate the hydrogen atoms linked to ammonium ions and isotropic refinements were then done. Crystal data for compound **1** are listed in Supplementary Table 1 in the supporting information. CCDC = 1814836 contains the supplementary crystallographic data discussed in this study. These data can be obtained free of charge from the Cambridge Crystallographic Data Centre via www.ccdc.cam.ac.uk/data_request/cif.

Supplementary Tables

Supplementary Table 1 │ Crystal data and structure refinement for compound 1

Empirical formula	$C_{12}H_{11}Cu_2N_3O_4S_2$
F_w	452.44
Temperature/K	200(2)
Crystal system	Orthorhombic
Space group	$Pna2_1$
a, Å	11.3797(5)
b, Å	3.8231(10)
c, Å	31.2186(14)
α (deg)	90
β (deg)	90
γ (deg)	90
V/Å^3	1358.19
Z	4
λ/Å	0.71073
D_{calc}(g cm^{-3})	2.213
μ/mm^{-1}	3.463
F(000)	904
Reflections collected	7449
Independent reflections	2620 (R_{int} = 0.0428)
Data/restraints/parameters	2620/23/224
Completeness to theta = 25.242°	99.8%
GOF	1.037
$^aR_1$, $^bwR_2$ [$I > 2\sigma(I)$]	0.0340, 0.0760
$^aR_1$, $^bwR_2$ (all data)	0.0483, 0.0838

$^aR_1 = \Sigma||F_0|-|F_c|| / \Sigma|F_0|$; $^bwR_2 = [\Sigma w(F_0{}^2-F_c{}^2)^2 / \Sigma w(F_0{}^2)^2]^{1/2}$

Supplementary Table 2 │ Comparison of electrical conductivity for compound 1

Compounds	Type	Conductivity ($S\ cm^{-1}$)	Activation Energy (eV)	References
$\{[Cu_2(6\text{-}Hmna)(6\text{-}mn)]NH_4\}_n$	crystal (4 probe)	10.96	0.006 (300 K)	this work
$Cu_3(HHTP)_2$	crystal (4 probe)	0.2	N.R.	4
$Fe_2(BDT)_3$	crystal (2 probe)	1.8	0.16	5
NNU-27	crystal (2 probe)	1.3×10^{-3}	N.R.	6
$Cd_2(TTFTB)$	crystal (2 probe)	2.9×10^{-4}	N.R.	7
$Zn_2(TTFTB)$	crystal (2 probe)	4×10^{-6}	N.R.	7
$Mn_2(TTFTB)$	crystal (2 probe)	8.6×10^{-5}	N.R.	7
$Co_2(TTFTB)$	crystal (2 probe)	1.5×10^{-5}	N.R.	7
$Cu_3(BHT)_2$	film (4 probe)	1580	0.00012 (40 K)	8
$Ni_3(HITP)_2$	film (van der Pauw)	40	N.R.	9
$TCNQ@Cu_3(BTC)_2$	film (4 probe)	0.07	0.041 (125–300 K)	10
$Pd_3(BHT)_2$	film (4 probe)	2.8×10^{-2}	N.R.	11
$Cu[Ni(pdt)_2]$ (I_2-doped)	film (2 probe)	1×10^{-4}	0.18	12
$Cu[Ni(pdt)_2]$	film (2 probe)	1×10^{-8}	0.49	12
$Ni_3(BHT)_2$	Microflake (van der Pauw)	160, 2.8	0.010	13
$Ni_3(HITP)_2$	pellet (2 probe)	2	N.R.	9
$Cu_3(HITP)_2$	pellet (2-probe)	0.2	N.R.	14
$(NBu_4)_2Fe_2(DHBQ)_3$	pellet (2 probe)	0.16	0.11 (70–300 K)	15
$Na_{0.9}(NBu_4)_{1.8}Fe_2(DHBQ)_3$	pellet (2 probe)	6.2×10^{-3}	0.18 (70–300 K)	15
$Ni_3(BHT)_2$	pellet (2 probe)	0.15	0.026	16
$Fe(1,2,3\text{-Triazolate})_2$	pellet (4 probe)	7.7×10^{-5}	N.R.	17
$Fe_2(DSBDC)$	pellet (2 probe)	3.9×10^{-6}	0.28 (200–420 K)	18
$Fe_2(DOBDC)$	pellet (2 probe)	3.2×10^{-7}	0.38 (200–420 K)	18
$Mn_2(DSBDC)$	pellet (2 probe)	2.5×10^{-12}	0.81 (320–420 K)	18
$Mn_2(DOBDC)$	pellet (2 probe)	3.9×10^{-13}	0.54 (210–420 K)	18
$Pt_3(HTTP)_2$	pellet (2-probe)	1×10^{-6}	N.R.	19
$Cu[Cu(PDT)_2]$	N.R.	6×10^{-4}	0.193 (200–400 K)	20

Supplementary Table 3 │ Calculation of the oxidation state of copper by the bond valence sum for 1

Coordinate bonds	R_o (A°)	R (A°)	b	$S = e^{\{(Ro-R)/b\}}$	$V = \sum S$
Cu(1)–S(1)	1.811	2.343	0.37	0.237	–
Cu(1)–N(1)	1.520	2.023	0.37	0.256	–
Cu(1)–S(1) × 3	–	–	–	0.711	–
(Cu(1)–S(1) × 3) + (Cu(1)–N(1))	–	–	–	–	0.967

R_o = the length of bond of unit valence, R = observed bond length, b = empirical constant, S = individual bond valance, V = sum of individual bond valance.

Supplementary Figures

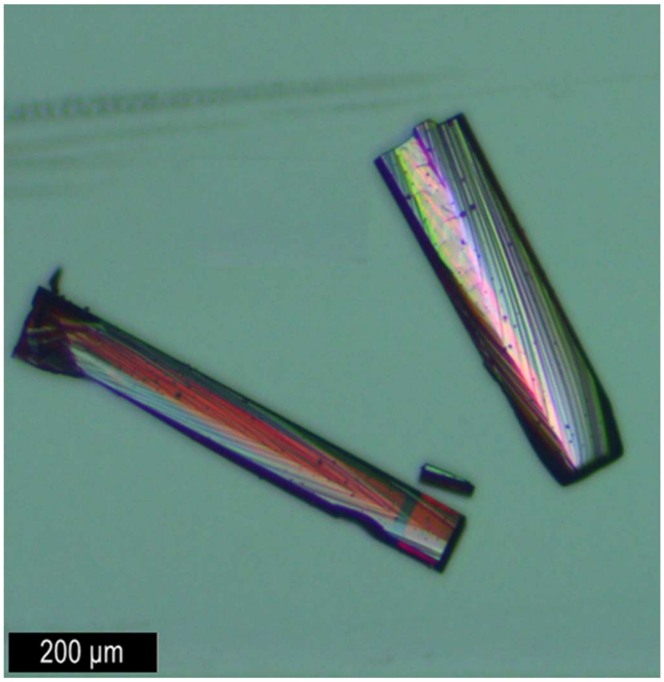

Supplementary Figure 1 │ Bright Field optical Image of 1. Bright field optical images of crystals of **1**.

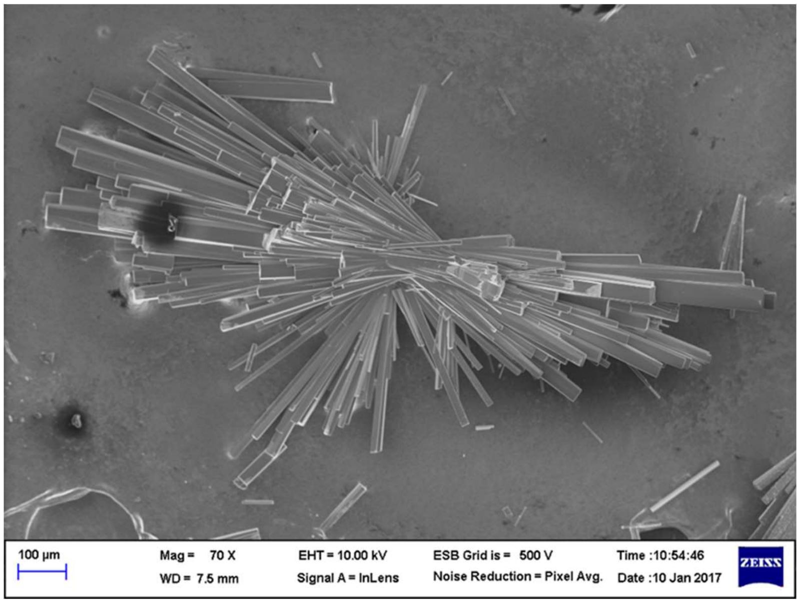

Supplementary Figure 2│ Scanning electron microscope image of 1. SEM image of compound **1**.

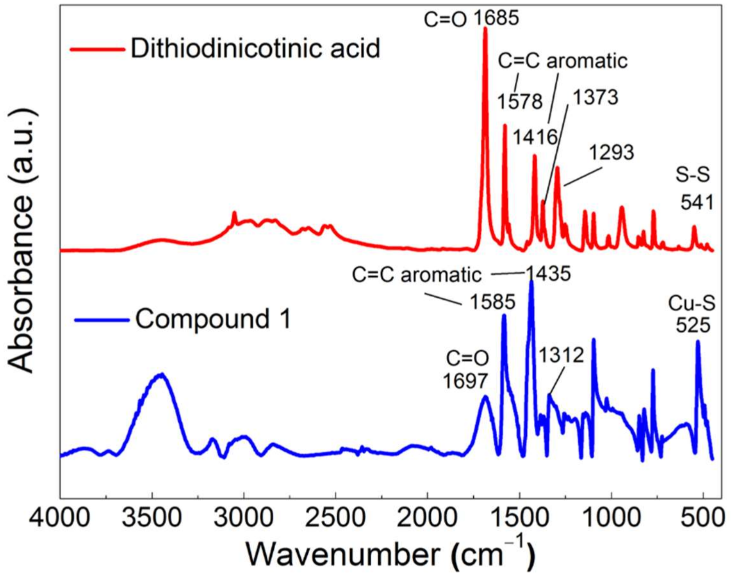

Supplementary Figure 3│ Fourier-Transform infrared spectrum of 1. FTIR spectra of 6,6′-dithiodinicotinic acid and compound **1**.

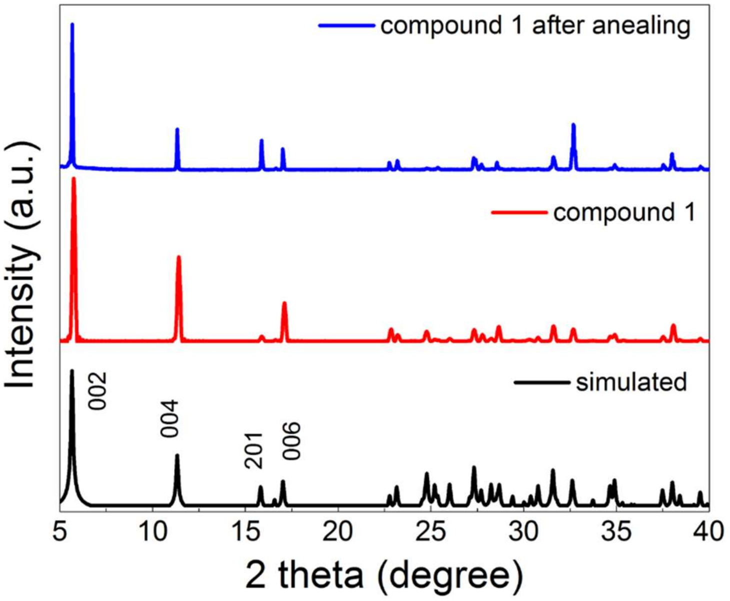

Supplementary Figure 4│ Powder X-ray Diffraction of 1. PXRD patterns of compound **1** before and after annealing at 100 °C.

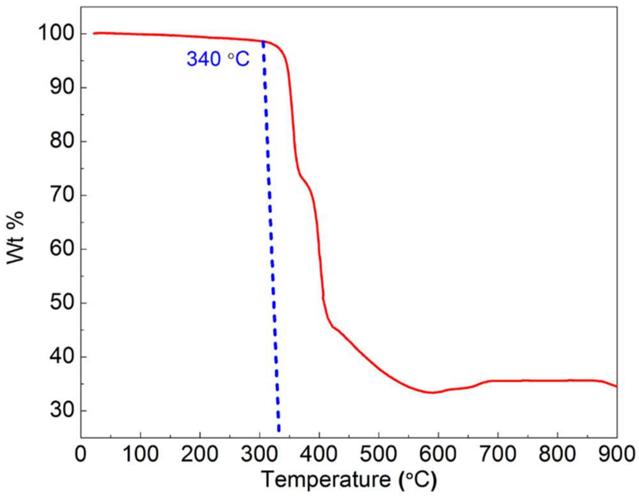

Supplementary Figure 5│Thermogravimetric analysis of compound 1. A thermogravimetric analysis (TGA) indicated that compound **1** is thermally stable at temperatures up to 340 °C. A TGA analysis was done for MOF **1** after the removal of surface adsorbed water by storing the crystals in desiccator with activated molecular sieves

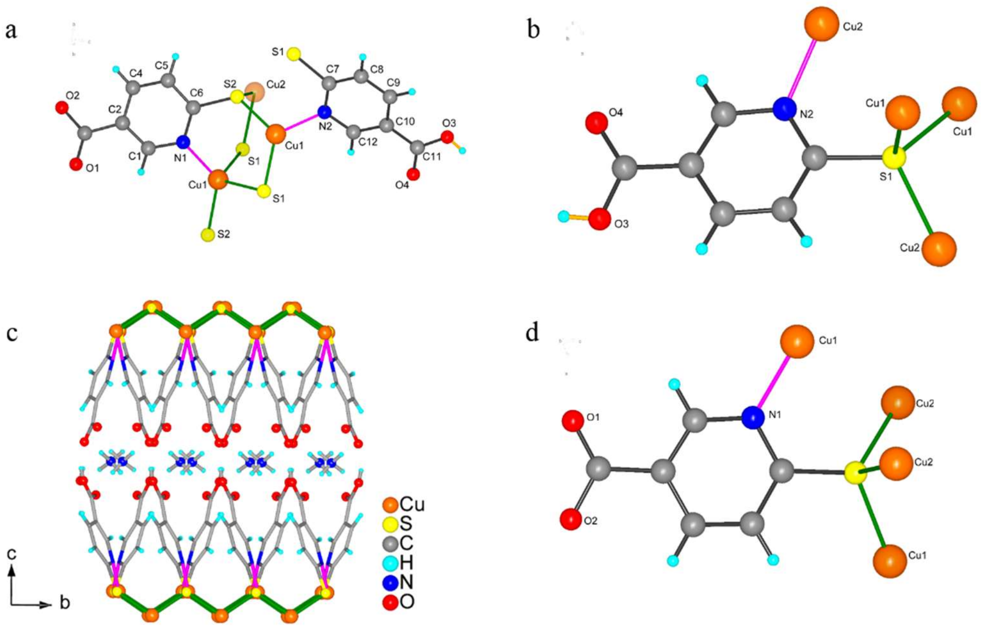

Supplementary Figure 6│Crystal structure of 1. (a) Coordination environment for **1**. (b) Coordination mode of 6-Hmna ligand (c) Perspective view of the crystal structure of **1** along the *a*-axis (Cu = orange, O = red, C = light grey, N = blue, S = yellow, H = cyan) (d) Coordination mode of 6-mn ligand.

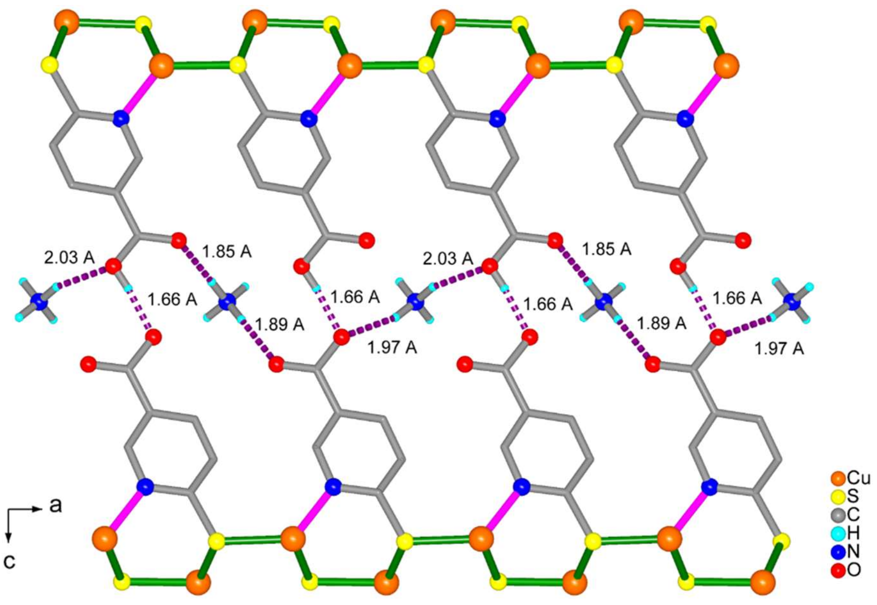

Supplementary Figure 7│ Hydrogen bonding in crystal structure of 1. Hydrogen bonding interactions between ammonium ions and ligands for compound **1**.

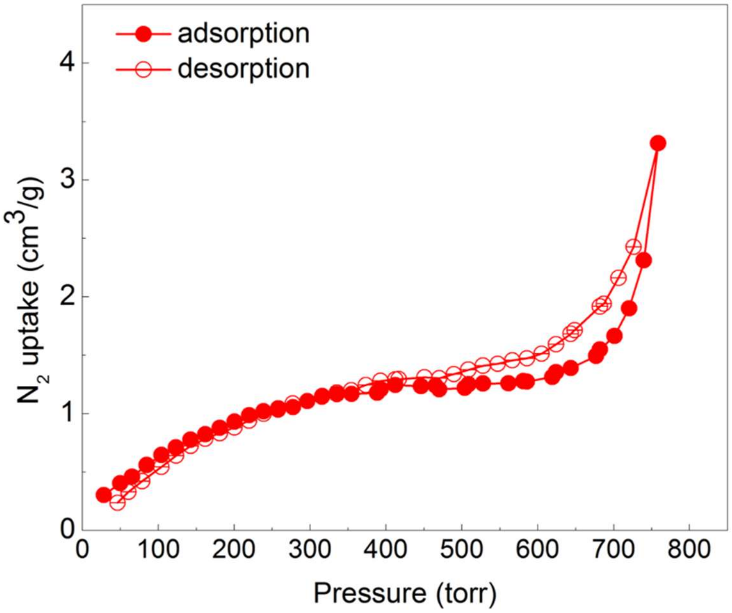

Supplementary Figure 8 | Brunauer-Emmett- Teller (BET) isotherm of 1. N_2 adsorption and desorption isotherms of compound **1** at 77 K.

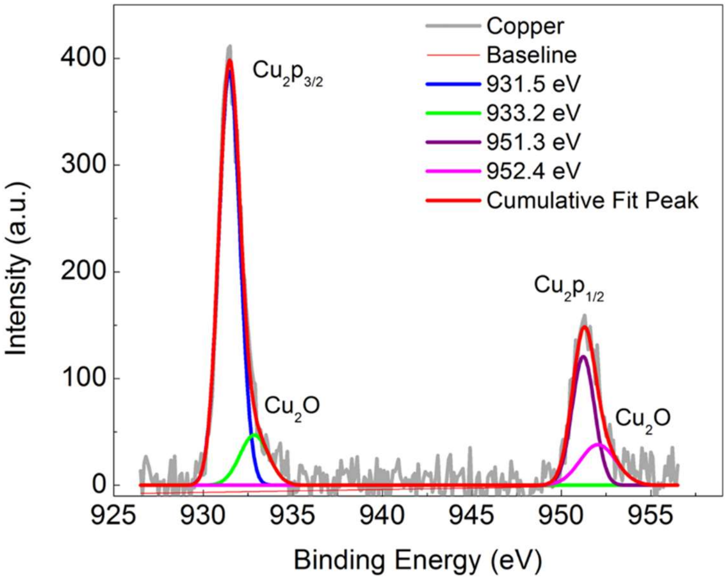

Supplementary Figure 9 | X-ray photoelectron spectrum of copper of 1. XPS spectrum of the Cu(I) ion of compound **1**.

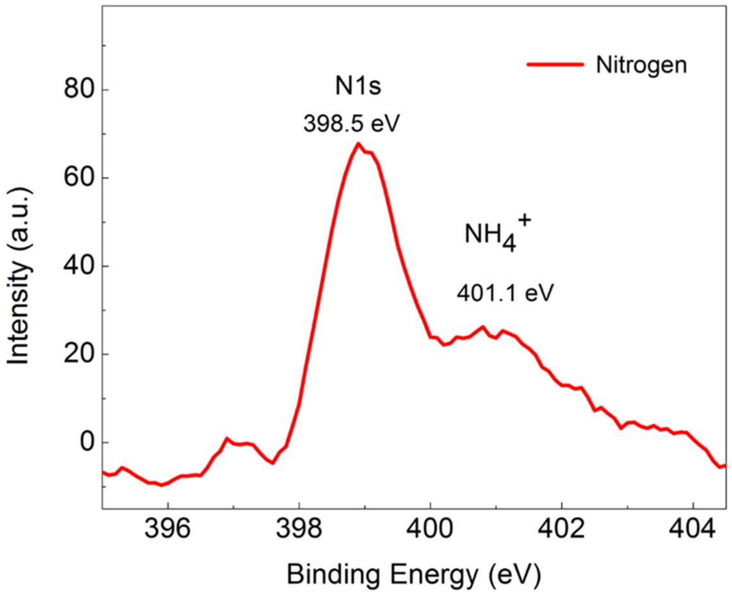

Supplementary Figure 10 | X-ray photoelectron spectrum of nitrogen of 1. XPS spectrum of the nitrogen atoms of compound **1**.

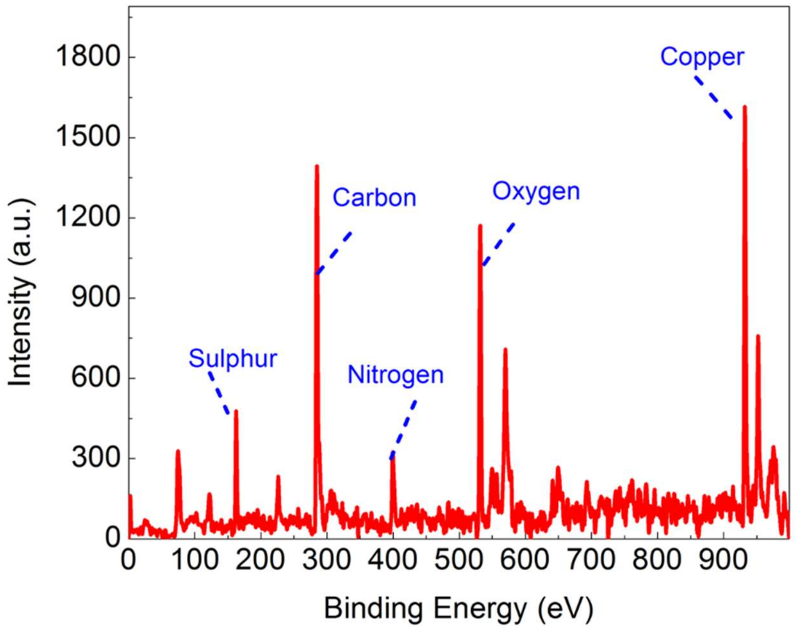

Supplementary Figure 11 | X-ray photoelectron spectrum of 1. Full XPS spectrum of **1**.

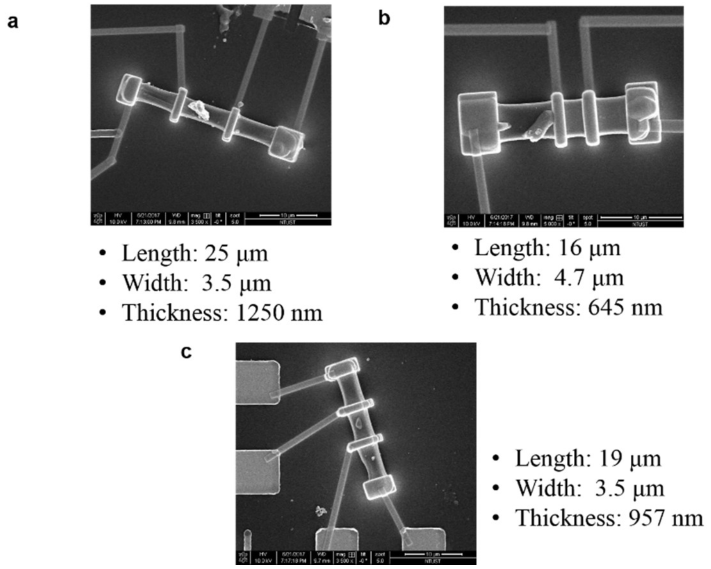

Supplementary Figure 12 | Scanning electron microscope image of device of 1. SEM images of fabricated devices containing different thicknesses of single crystals of **1**.

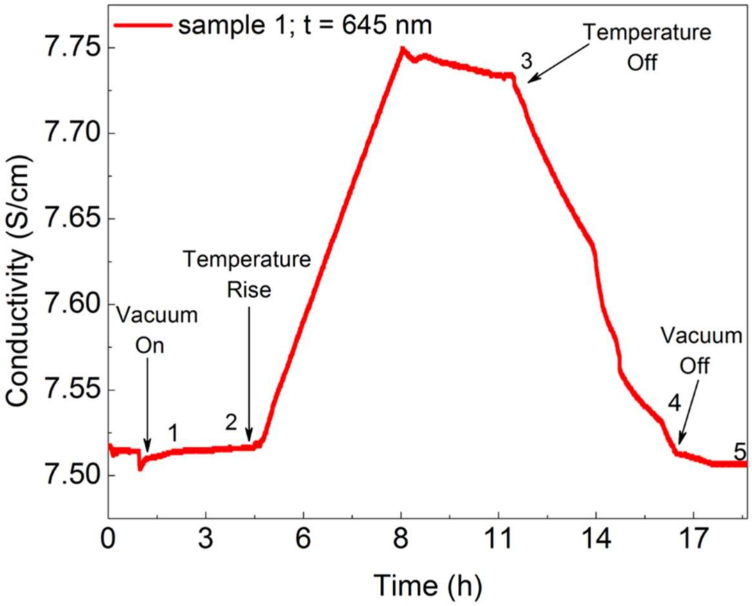

Supplementary Figure 13 | Temperature dependent conductivity of 1 with time. Temperature dependent conductivities of the copper MOF **1** when stored over a period of 18 hours in a vacuum after annealing at 50 °C.

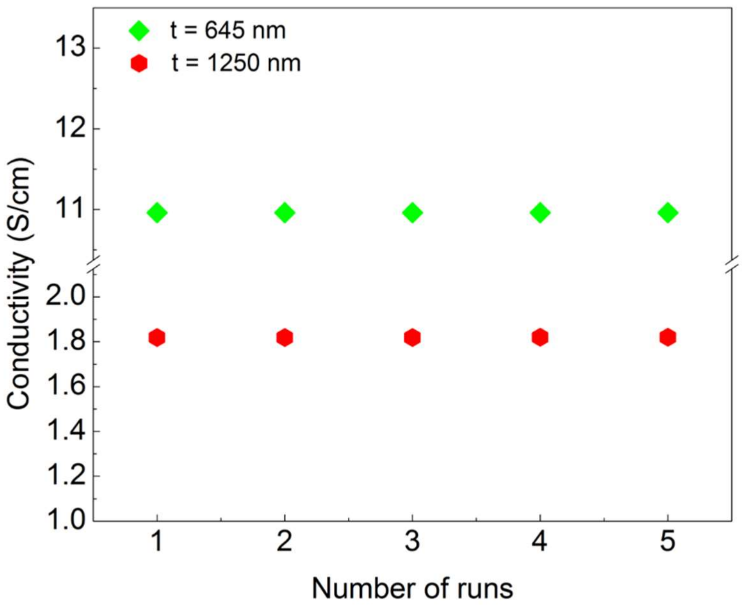

Supplementary Figure 14 | Electrical conductivity with the number of runs of 1. Electrical conductivities of different thicknesses (t) of **1** with different run numbers with a low standard deviation (0.00065).

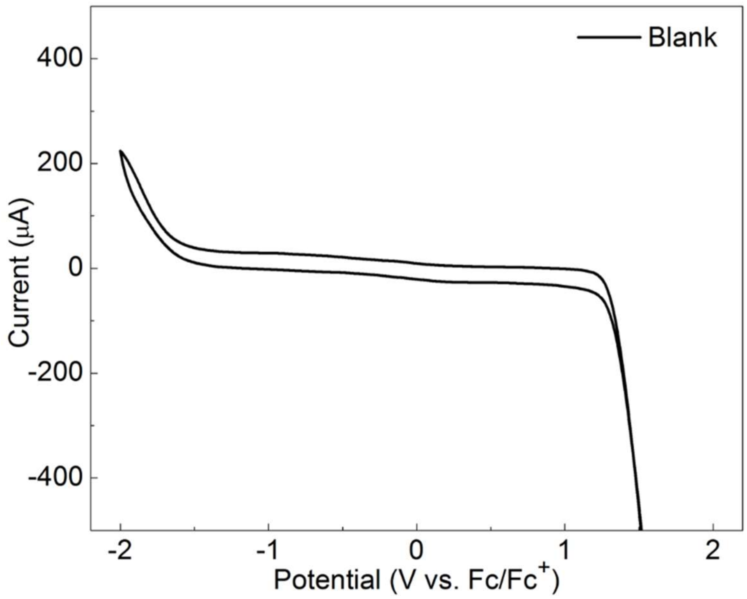

Supplementary Figure 15 | Blank cyclic voltammetry. Blank run of cyclic voltammetry of **1**.

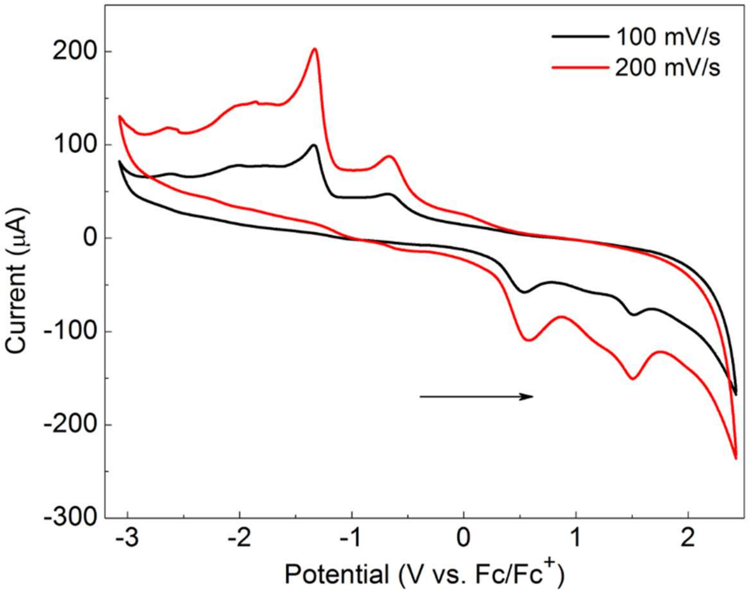

Supplementary Figure 16│ Cyclic voltammetry of 1. Cyclic voltammograms of compound **1**.

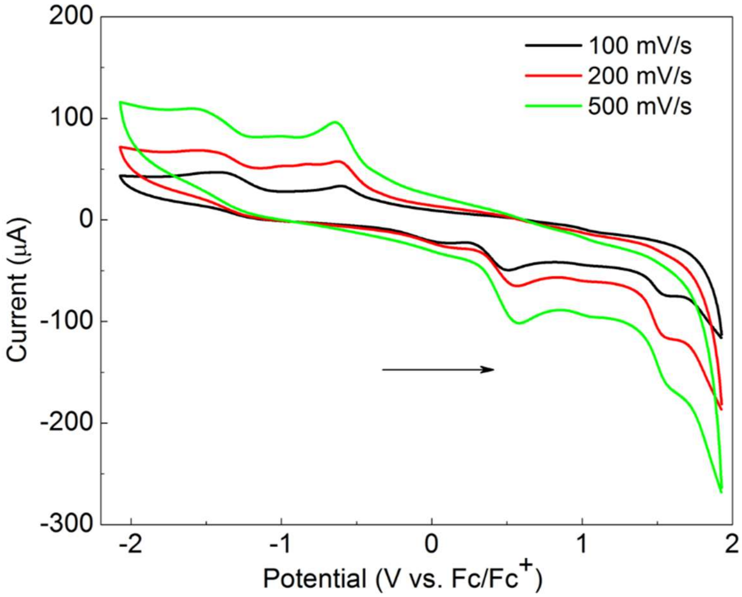

Supplementary Figure 17│ Cyclic voltammetry of ligand. Cyclic voltammograms of the 6-mercaptonicotinic acid (6-Hmna) ligand.

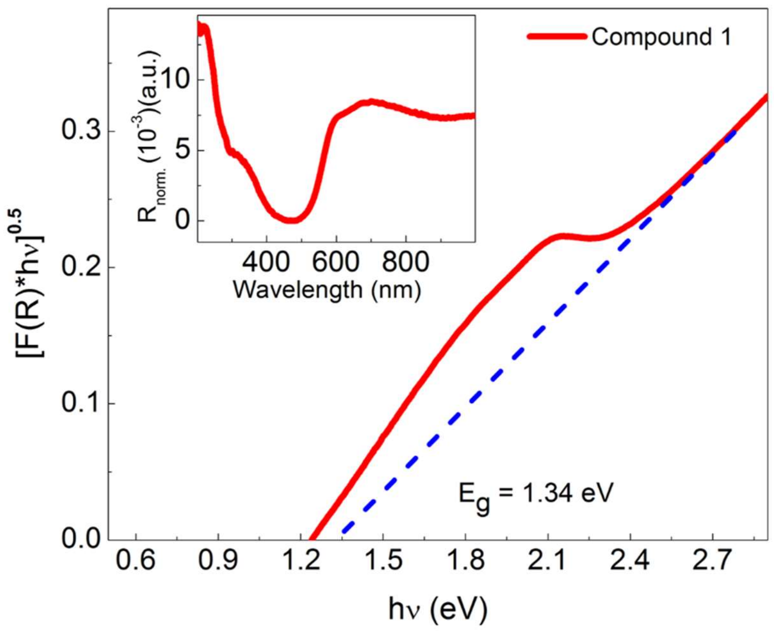

Supplementary Figure 18 │ Kubelka Munk function versus band gap of 1. Kubelka Munk function of **1** and the inset showing normalized reflectance.

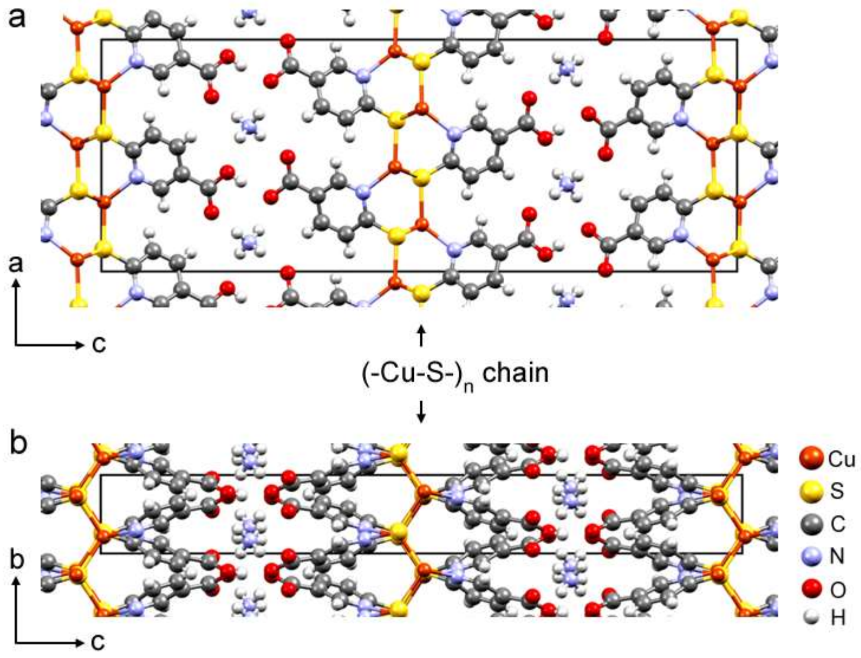

Supplementary Figure 19 │ Crystal structure of 1. (a) Top and (b) side views of the periodic unit cell of **1**.

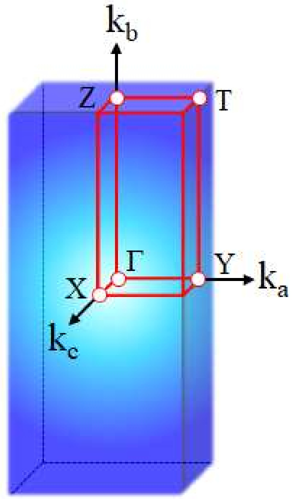

Supplementary Figure 20│ Primitive cell Brillouin zone of 1. A view of the Brillouin zone of a primitive cell with high symmetry lines of **1**.

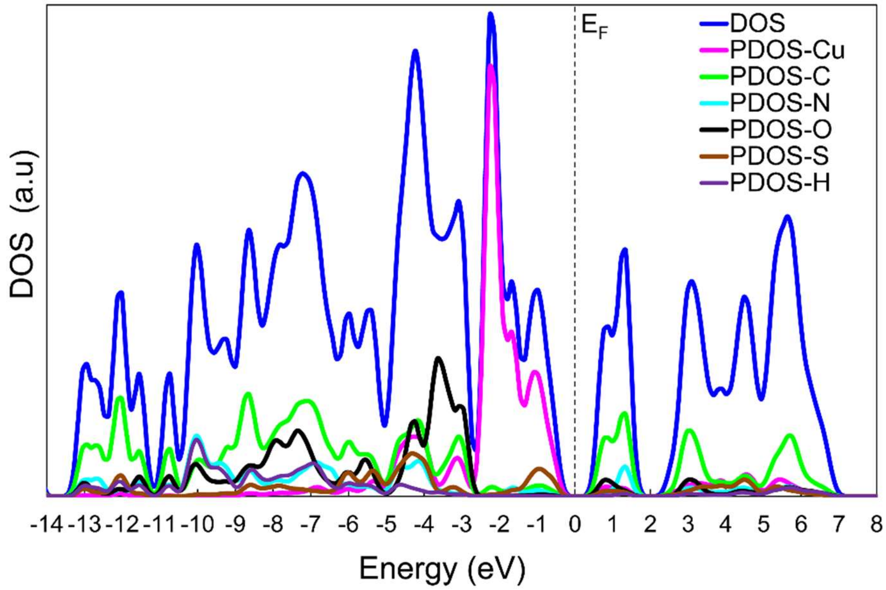

Supplementary Figure 21│Density of states and partial density of states of 1. Total (blue area) and partial density of states (other colored areas) of **1**. The dashed line at zero energy represents the Fermi level (E_F).

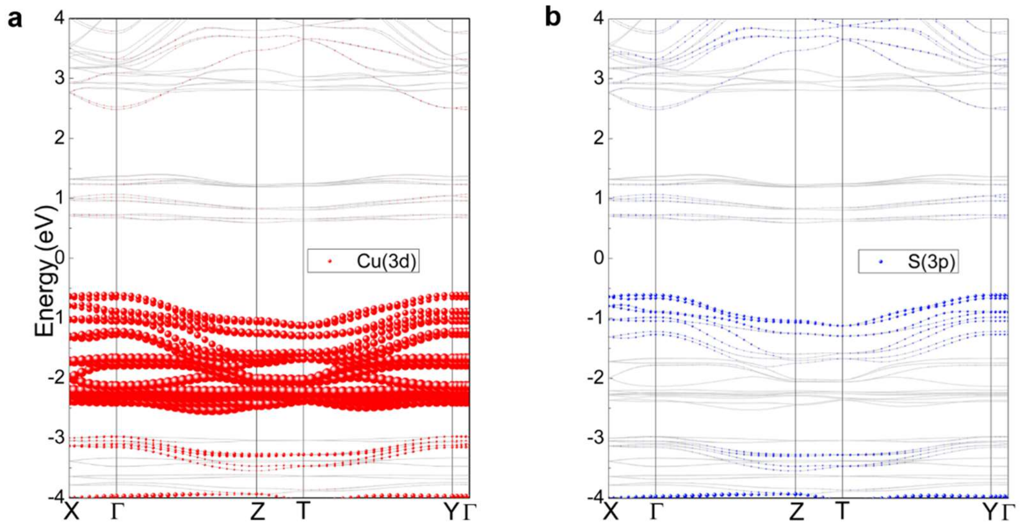

Supplementary Figure 22 | The orbital-decomposed band structure of 1. Here red and blue spheres represent the 3d orbitals of copper (a) and the 3p orbitals of sulfur (b), respectively. The diameters of the spheres are proportional to the magnitude of the orbital contribution. Importantly, the orbital-decomposed band structures clearly indicate that the VBM primarily originates from the hybridization of the 3d orbitals of copper and the 3p orbitals of sulfur (note that the s- and p-orbitals of Cu, as well as s-orbital of S, are not displayed here since their contribution to the VBM is negligible).

Supplementary References

1 Farrugia, L. J. *J. Appl. Crystallogr.* **32**, 837-838, (1999).

2 Spek, A. L. *J. Appl. Crystallogr.* **36**, 7-13, (2003).

3 Sheldrick, G. M. *A Short History of SHELX*, *Acta Cryst*. **A64**, 112-122, (2008).

4 Hmadeh, M. *et al.* New Porous Crystals of Extended Metal-Catecholates. *Chem. Mater.* **24**, 3511-3513, (2012).

5 Xie, L. S. *et al.* Tunable Mixed-Valence Doping toward Record Electrical Conductivity in a Three-Dimensional Metal–Organic Framework. *J. Am. Chem. Soc.* **140**, 7411-7414, (2018).

6 Chen, D., Xing, H., Su, Z. & Wang, C. Electrical conductivity and electroluminescence of a new anthracene-based metal–organic framework with π-conjugated zigzag chains. *Chem. Commun.* **52**, 2019-2022, (2016).

7 Park, S. S. *et al.* Cation-Dependent Intrinsic Electrical Conductivity in Isostructural Tetrathiafulvalene-Based Microporous Metal–Organic Frameworks. *J. Am. Chem. Soc.* **137**, 1774-1777, (2015).

8 Huang, X. *et al.* A two-dimensional π–d conjugated coordination polymer with extremely high electrical conductivity and ambipolar transport behaviour. *Nat. Commun.* **6**, 7408, (2015).

9 Dong, R. *et al.* Large-Area, Free-Standing, Two-Dimensional Supramolecular Polymer Single-Layer Sheets for Highly Efficient Electrocatalytic Hydrogen Evolution. *Angew. Chem. Int. Ed.* **54**, 12058-12063, (2015).

10 Talin, A. A. *et al.* Tunable Electrical Conductivity in Metal−Organic Framework Thin-Film Devices. *Science* **343**, 66-69, (2014).
11 Pal, T. *et al.* Interfacial Synthesis of Electrically Conducting Palladium Bis(dithiolene) Complex Nanosheet. *ChemPlusChem* **80**, 1255-1258, (2015).
12 Kobayashi, Y., Jacobs, B., Allendorf, M. D. & Long, J. R. Conductivity, Doping, and Redox Chemistry of a Microporous Dithiolene-Based Metal−Organic Framework. *Chem. Mater.* **22**, 4120-4122, (2010).
13 Kambe, T. *et al.* Redox Control and High Conductivity of Nickel Bis(dithiolene) Complex π-Nanosheet: A Potential Organic Two-Dimensional Topological Insulator. *J. Am. Chem. Soc.* **136**, 14357-14360, (2014).
14 Sheberla, D. *et al.* High Electrical Conductivity in Ni_3(2,3,6,7,10,11-hexaiminotriphenylene)$_2$, a Semiconducting Metal–Organic Graphene Analogue. *J. Am. Chem. Soc.* **136**, 8859-8862, (2014).
15 Darago, L. E., Aubrey, M. L., Yu, C. J., Gonzalez, M. I. & Long, J. R. Electronic Conductivity, Ferrimagnetic Ordering, and Reductive Insertion Mediated by Organic Mixed-Valence in a Ferric Semiquinoid Metal–Organic Framework. *J. Am. Chem. Soc.* **137**, 15703-15711, (2015).
16 Kambe, T. *et al.* π-Conjugated Nickel Bis(dithiolene) Complex Nanosheet. *J. Am. Chem. Soc.* **135**, (2013).
17 Gándara, F. *et al.* Porous, Conductive Metal-Triazolates and Their Structural Elucidation by the Charge-Flipping Method. *Chem. Eur. J.* **18**, 10595-10601, (2012).
18 Sun, L., Hendon, C. H., Minier, M. A., Walsh, A. & Dincă, M. Million-Fold Electrical Conductivity Enhancement in Fe_2(DEBDC) versus Mn_2(DEBDC) (E = S, O). *J. Am. Chem. Soc.* **137**, 6164-6167, (2015).
19 Cui, J. & Xu, Z. An electroactive porous network from covalent metal–dithiolene links. *Chem. Commun.* **50**, 3986-3988, (2014).
20 Takaishi, S. *et al.* Electroconductive Porous Coordination Polymer Cu[Cu(pdt)$_2$] Composed of Donor and Acceptor Building Units. *Inorg. Chem.* **48**, 9048-9050, (2009).

.
